# Topoisomerase II is regulated by translationally controlled tumor protein for cell survival during organ growth in *Drosophila*

**DOI:** 10.1038/s41419-021-04091-y

**Published:** 2021-08-27

**Authors:** Dae-Wook Yang, Jung-Wan Mok, Stephanie B. Telerman, Robert Amson, Adam Telerman, Kwang-Wook Choi

**Affiliations:** 1 Department of Biological Sciences, Korea Advanced Institute of Science and Technology (KAIST), Daejeon, 34141 Korea; 2 Department of Genetics, University of Cambridge, Cambridge, CB2 3EH UK; 3 Institut Gustave Roussy, Unité Inserm U981, Bâtiment B2M, 114 rue Édouard-Vaillant, 94805 Villejuif, France

**Keywords:** Cell growth, Organogenesis

## Abstract

Regulation of cell survival is critical for organ development. Translationally controlled tumor protein (TCTP) is a conserved protein family implicated in the control of cell survival during normal development and tumorigenesis. Previously, we have identified a human Topoisomerase II (TOP2) as a TCTP partner, but its role in vivo has been unknown. To determine the significance of this interaction, we examined their roles in developing *Drosophila* organs. *Top2 RNAi* in the wing disc leads to tissue reduction and caspase activation, indicating the essential role of Top2 for cell survival. *Top2 RNAi* in the eye disc also causes loss of eye and head tissues. *Tctp RNAi* enhances the phenotypes of *Top2 RNAi*. The depletion of Tctp reduces Top2 levels in the wing disc and vice versa. Wing size is reduced by Top2 overexpression, implying that proper regulation of Top2 level is important for normal organ development. The wing phenotype of *Tctp RNAi* is partially suppressed by Top2 overexpression. This study suggests that mutual regulation of Tctp and Top2 protein levels is critical for cell survival during organ development.

## Introduction

TCTP is a growth control protein, initially found as P23 in murine and human tumor cells [1, 2]. Evolutionarily conserved TCTP family proteins are involved in diverse biological processes, including growth, immune response, and cytoskeletal changes [3–6]. Abnormal regulation of TCTP has been implicated in the pathogenesis of growth-related diseases. Human TCTP is upregulated in various types of cancer cells or tissues [7–9]. Furthermore, tumors can be reverted by reducing the TCTP function, implying its critical role in cancer [10, 11].

TCTP is also involved in the regulation of apoptosis [12–17]. Consistent with the anti-apoptotic function of TCTP, its overexpression can induce chemoresistance to cancer cells or tissues treated with anti-cancer drugs including etoposide [18–22]. Since the primary target of etoposide is Topoisomerase II (TOP2) [23–25], it is an intriguing question whether the anti-apoptotic function of human TCTP is functionally related to TOP2.

Top2 cuts double-strand DNA to relax its tangles during replication and transcription. In vertebrates, there are two subclasses of type II topoisomerases, TOP2A and TOP2B. An important biological function of TOP2 is to regulate tissue growth since *top2* mutants in zebrafish and mice show growth defects [26–28]. Like TCTP, a human TOP2 paralog TOP2A is highly overexpressed in cancer cells [29–31], and its knockdown suppresses the invasive capacity of the cancer cells [30, 32, 33]. These findings suggest that TCTP and TOP2 might play similar roles in growth control. However, it is unknown whether there is a functional relationship between TCTP and TOP2 in vivo.

*Drosophila* has been used as a powerful model organism to study in vivo functions of TCTP. Genetic analysis has shown that *Drosophila* TCTP (Tctp) is required for organ development by regulating TOR signaling [3, 34], cell cycle [4, 35], and cell junctions [36]. It also regulates DNA damage response [37], tissue homeostasis in intestinal stem cells [38], and global gene expression [39]. These studies indicate that Tctp has a multitude of functions in different subcellular compartments to regulate growth-related processes.

*Drosophila* has only one gene for Top2 that is expressed throughout development. Top2 is involved in several processes, including chromosome condensation [40], chromatin remodeling [41], pairing or separation of chromosomes [42, 43], dosage compensation [44], and cell cycle regulation [45–47]. Given these various roles of Top2 during development, *Top2* null mutants show pupal lethality with abnormal growth of larval brain and imaginal discs [48]. Although Top2 is required for mitosis in the larval brain [47], specific roles of Top2 in imaginal disc development have not been characterized.

We initially discovered the human TCTP-TOP2 interaction in a yeast two-hybrid screen for TCTP binding proteins [17]. In this study, we investigated the functional relationships between Tctp and Top2 in *Drosophila* organ development. Our data suggest that Tctp and Top2 regulate each other to maintain their levels for cell survival during organ development.

## Materials and methods

### Fly stocks and genetics

*Drosophila melanogaster* stocks were cultured in a standard cornmeal medium. Most genetic crosses were carried out at 25 °C unless stated otherwise. *w*^*1118*^ was used as the wild-type control. GAL4 lines are as follows: *en-GAL4*, *nub-GAL4*, and *ey-GAL4*. The following lines were from Bloomington Drosophila Stock Center (BDSC), Vienna Drosophila Resource Center (VDRC), or National Institute of Genetics (NIG): *Top2 RNAi* (BDSC 31342, VDRC v30625, v330177, and NIG10223R-2), and *UAS-Diap1* (BDSC 6657). *Tctp RNAi* was described [3]. *Top2*^*Suo1*^ mutant was obtained from Dr. Silvia Bonaccorsi [47].

### Antibodies, immunostaining, and imaging

Imaginal discs were dissected from third instar larvae in cold phosphate-buffered saline (PBS) (pH 7.4). Samples were fixed in PLP (2–4% paraformaldehyde, 10 mM sodium periodate, 75 mM lysine, and 35 mM sodium phosphate buffer or PBS, pH 7.4) for 15 min at room temperature (RT). Discs samples were washed with PBS for 5 min two times. Samples were treated with blocking buffer (0.3% Triton X-100, 5% normal goat serum and 0.08% NaN_3_ in PBS, or 0.3% Triton X-100, 0.5% BSA, 0.01% NaN_3_ in PBS) for 2 h at 4 °C or 40 min at RT, and incubated with primary antibodies diluted in washing buffer (0.3% Triton X-100 in PBS) overnight at 4 °C. Primary antibodies were: rabbit anti-cleaved Death caspase-1 (Dcp-1) (Asp216) (Cell Signaling Technology 9578, 1:100), rabbit anti-Top2 (gift from Dr. Donna Arndt-Jovin, 1:2000) [45], and rabbit anti-Tctp (1:100) [3].

After incubation with primary antibodies, samples were rinsed with washing buffer for 15 min six times and treated with secondary antibodies (Jackson immune research laboratories,1:200) in washing buffer for 2 h at RT. Samples were rinsed with washing buffer for 10 min six times and PBS for 5 min two times. For DNA staining, samples were treated with 4,6 Diamidine-2-phenylinddedin (DAPI) (Boehringer Mannheim, Germany) at 1:1000 during the last washing in washing buffer. Samples were mounted with vectashield (H-1000, Vector Laboratories, USA) and imaged using a confocal microscope (ZEISS LSM710 or 780). The ZEN program was used for image analysis.

Adult wings were mounted using a wing mounting solution (1:1 mix of Canada balsam-sigma C1795 and methyl salicylate-sigma M6725) and imaged using a light microscope (ZEISS Axio Imager M2) with Axio vision Rel4.8 program. Adult eye or pupa pictures were taken using a light microscope (ZEISS KL 1500 LCD Axio cam MRC, stemi 2000-C) with Axio vision Rel4.8 program.

### Western blot

Protein samples were separated in SDS-PAGE gel with electrophoresis buffer and transferred to the PVDF membrane (Immobilon-P) after 100% methanol activation. Blots were incubated in a blocking solution containing 5% skim milk (232100 from BD Difco) or 3–5% BSA (BSA-BSH from RMBIO) in TBST (10 mM Tris-Cl pH 7.5, 150 mM NaCl, and 0.1% Tween-20) for 30 min at RT. Blots were incubated in the blocking solution containing primary antibodies at 4 °C overnight. Primary antibodies were: rabbit anti-Top2 (1:5000) [45], rabbit anti-Tctp (1:2000) [3], and mouse anti-β-Tubulin (DSHB, E7, 1:10000).

After incubation with primary antibodies, samples were washed for 15 min four times using washing buffer TBST, and incubated with secondary antibodies (Jackson immune research laboratories, 1:10000) for 1 h at RT, and washed for 15 min four times. Membranes were incubated in ECL solution (Prod #34095, Prod #34080, or Prod #34580, Thermo Fisher Scientific) and exposed to X-ray film.

### S2 cell culture

S2 cell line (from DGRC) was cultured at 24 °C. Complete medium is composed of serum-free media (Gibco express five SFM, Thermo Fisher Scientific) with 200 mM L-Glutamine and antibiotics (penicillin and streptomycin) as described in the Thermo Fisher Scientific protocol.

### Transgene construction

A DNA fragment encoding Top2 from *pFlc1-Top2* (DGRC RE49802) was inserted into the *pUAST-attB* vector for making the *pUAST-attB*-*Top2* construct. According to DGRC, *Top2 cDNA* has a deletion mutation in 304–364, point nonsense mutation; stop codon in 3802–3601. However, our analysis detected a deletion mutation at 333, and stop codon at 3817. Therefore, we corrected these mutations in *pFlc1-Top2*.

PCR was done using Prime star HS DNA polymerase (Takara, R010A) with primers. PCR products were separated in 1% agarose gel (Seakem LE agarose for gel electrophoresis, Lonza, 50004), purified using an Accuprep gel purification kit (Bioneer, Korea), and sequenced (Solgent, Korea).

### Generation of transgenic fly lines

The corrected *Top2* cDNA described above was used to generate *pUAST-Top2*. Transgenic lines (*UAS-Top2-1 to UAS-Top2-5*) were generated by BestGene (USA) using *P[acman] attP* strain; *PBac{yellow[+]-attP-3B}VK00002* (BDSC 9723, 2^nd^ chromosome).

### Synthesis of double-strand RNA

cDNAs for *pBluescript SK(-), Tctp*, and *Top2* were used as templates to generate double-strand RNA (dsRNA). Primers were synthesized (Bioneer, Korea). Primer target sites for *Control dsRNA*, *Tctp dsRNA* (same as *UAS-Tctp RNAi* described in ref. [3] except that the Xba Ι restriction site was deleted), and *Top2 dsRNA* were selected as described [3, 37, 49]. The sequences of primers are as follows:

*Control dsRNA* F: TAATACGACTCACTATAGG ATCGATAAGCTTGATATCGAATTC

*Control dsRNA* R:

TAATACGACTCACTATAGG GCACCGCCTACATACCTCGCTCTG

*Tctp dsRNA* F: TAATACGACTCACTATAGG TGTTTGCCGACACCTACAAG

*Tctp dsRNA* R: TAATACGACTCACTATAGG CCGTCGCAGTCCATAGATTC

*Top2 dsRNA* F: TAATACGACTCACTATAGGG TTTGCCAGAGCGATATCTC

*Top2 dsRNA* R: TAATACGACTCACTATAGGG CCATAGTGGCTCGATCTTTT

In vitro transcription was carried out with dsRNA templates using EZ™ T7 High Yield In Vitro Transcription Kit (Enzynomics, EZ027S) at 37 °C overnight. Next, the RNA mixtures were treated with DNase Ι (Amplification grade, Thermo Fisher Scientific, 18068015) for 30 min at 37 °C. Samples were purified using phenol-chloroform isoamyl alcohol mixture (Sigma, 77619), and kept at −80 °C. In total, 30 µg dsRNA was transfected into S2 cells and incubated at 24 °C. dsRNA transfection was performed using the protocol of the *Drosophila* RNAi screening center (DRSC) at Harvard medical school.

### Real-Time PCR

*Drosophila* S2 cells were harvested after 4 days of treatment with dsRNA. Total RNA was extracted using TRIzol (Invitrogen, 15596026) following the manufacturer’s instructions. cDNA synthesis and the removal of genomic DNA (gDNA) were carried out using PrimeScript RT Reagent Kit with gDNA Eraser (Takara, RR047A). Real-time PCR experiments were conducted in triplicates or quadruplicates and analyzed using a C1000 Touch^TM^ Thermal Cycler (Bio-Rad, USA), CFX96^TM^ Real-Time system (Bio-RAD, USA) with QuantiNova SYBR Green PCR Kit (Qiagen, 208054). PCR reactions were initially incubated at 50 °C for 10 min, followed by denaturation for 15 min at 95 °C. After the pre-treatment, reactions were subjected to the following thermal cycling conditions: 40 cycles of denaturation at 95 °C for 15 s and annealing at 55 °C for 30 s with an extension at 72 °C for 30 s. After cycling, melting curve analyses were performed to check the existence of non-specific amplification and primer-dimers formation. Experiments were repeated 3 times using independently cultivated cells. All primers were purified with the Bio-RP scale and synthesized (Bioneer, Korea). Following primers with high primer efficiency (>90%) were used for amplification:

GAPDH1 F: CCACTGCCGAGGAGGTCAACTAC

GAPDH1 R: ATGCTCAGGGTGATTGCGTATGC

Tctp #1 F: GTTTCGCAGTGTTCCCGGTC

Tctp #1 R: TGGTGATTGGCTTGTCGGGA

Tctp #2 F: GGTCGTTTCGCAGTGTTCCC

Tctp #2 R: ATTGGCTTGTCGGGAGTCGG

Top2 #1 F: ACGGATACGGAGCGAAGC

Top2 #1 R: GAAGTCCTTGATCTGCACATC

Top2 #2 F: GCTTCACCGTTGAGACTGC

Top2 #2 R: GAAGTCCTTGATCTGCACATC

### Quantification and statistical analysis

Wing and eye sizes were measured using the Image J program (NIH, USA). Wing, eye, and pupa images were taken from representative samples with average size. Statistical analysis of phenotypes was performed using Prism 8 (GraphPad, USA). ‘n’ is the number of wings (or eyes) used to measure wing size. Sample mean (x̅) and sample standard deviation (s) were used when samples were selected from a population. ‘n’, ‘x̅’, and ‘s’ are similarly used in all Figures. Population mean (m) and population standard deviation (σ) were used when samples were not selected. We used an unpaired two-tailed student t-test with standard deviation (SD). If the SD of the two groups were significantly different based on *F*-test, we used Welch’s correction. Significance was defined by the rule of Graph pad program, n.s., not significant (*P* > 0.05), *P < 0.05, **P < 0.01, ***P < 0.001, and ****P < 0.0001. Stacked column graphs were made by Prism 8 (GraphPad, USA). Graph values were adjusted by using the fraction of the total analysis tool available in Prism 8.

## Results

### Knockdown of Top2 causes reduced wing growth

To characterize the role of Top2 in imaginal disc development, we used tissue-specific knockdown by RNAi using the GAL4-UAS method [50]. For Top2 knockdown, several *Top2 RNAi* lines that target the coding strand of *Top2* gene were utilized [51].

**Fig. 1:**
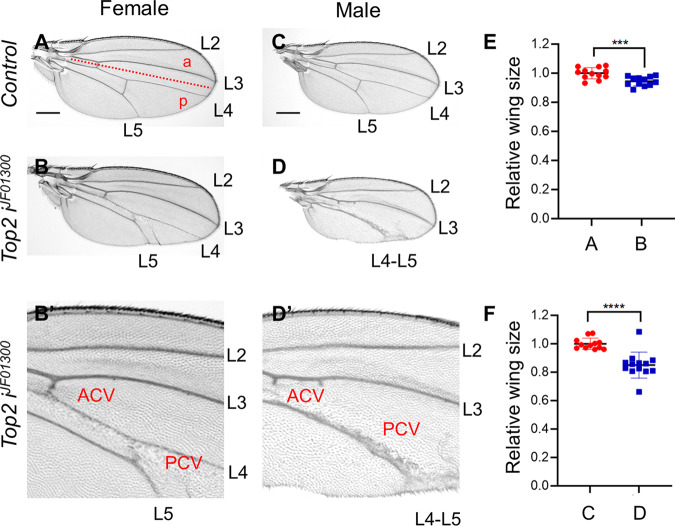
Knockdown of Top2 causes reduced wing growth. **A**, **B** Knockdown of Top2 reduces wing size in females. The A/P boundary is indicated by a dashed line. PCV and ACV are posterior and anterior crossvein, respectively. L2-L5 are longitudinal veins. (**A**) *en* > *GFP/+* (*n* = 12, x̅ ± s = 1 ± 0.039), (**B**’) *en* > *GFP* > *Top2 RNAi*^*JF01300*^ (*n* = 12, x̅ ± s = 0.945 ± 0.031). Scale bars are 300 µm. (**B**′) An enlarged view of (**B**). Knockdown of Top2 shows defects in the region between L3 and L5 veins in the female wing. (**C**, **D**) Knockdown of Top2 reduces wing in male. (**C**) *en* > *GFP/+* (*n* = 12, x̅ ± s = 1 ± 0.039), (**D**) *en* > *GFP* > *Top2 RNAi*^*JF01300*^ (*n* = 13, x̅ ± s = 0.850 ± 0.092). (**D**′) An enlarged view of (**D**). Knockdown of Top2 shows loss of wing area including L4 in males. The distal region of L4 and 5 veins are thicker than normal. (**E**) Quantification of relative wing sizes in (**A**, **B**). (**F**) Quantification of relative wing sizes in (**C**, **D**). Statistical analysis in **E**, **F** by unpaired two-tailed student *t*-test, ***P < 0.001, ****P < 0.0001. Error bars in (**E**, **F**) are SD. Scale bars are 300 μm (**A**, **C**) and 100 μm (**B**′, **D**′).

**Fig. 2:**
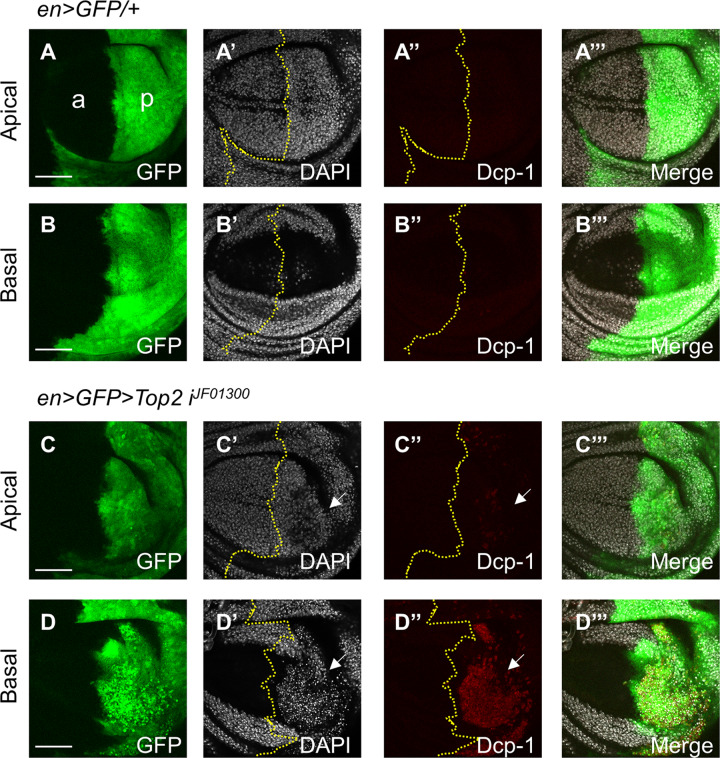
*Top2 RNAi* increases cell death. **A**–**B**’’’ *en* > *GFP/+* shows no change in the DAPI and Dcp-1 staining in the posterior region at 29 °C. **A**–**A**’’’ Apical section. **B**-**B**’’’ Basal section. Staining for GFP, DAPI, and Dcp-1 is as indicated in each panel. Yellow dotted lines in (**A**’–**D**’’) indicate the A/P boundary. Scale bars in (**A**–**D**) are 50 µm. a anterior, p posterior. **C**–**D**’’’ *en* > *GFP* > *Top2 RNAi*^*JF01300*^ shows increased Dcp-1 staining in the posterior region at 29 °C (3/3 discs; 100%). **C**–**C**’’’ Apical section. Dcp-1 staining is weakly increased (White arrow) (**C**”). **D**–**D**’’’ Basal section. Dcp-1 staining is strongly increased (White arrow in **D**"). DAPI staining is decreased (White arrow) in the posterior apical region (**C**’) while it is accumulated (White arrow) in the posterior basal region (**D**’).

First, *engrailed*-*GAL4* (*en-GAL4*) was used to express *Top2 RNAi* in the posterior compartment of the wing disc (‘*en* > *Top2 RNAi*’ in short). Knockdown of Top2 by three RNAi lines (*Top2 i*^*GD4570*^*, Top2 i*^*VSH330177*^, and *Top2 i*^*10223R-2*^) led to pupal lethality in both males and females at 25 °C. In contrast, *en* > *Top2 i*^*JF01300*^ flies were viable and showed growth defects in the wing. At 25 °C, female wings were slightly reduced (5.5 ± 3.1%) (Fig. 1A, B, E). In particular, the region between L4 and L5 in the posterior domain was significantly reduced with the near-complete loss of the posterior crossvein (PCV) (Fig. 1B’). Interestingly, such wing defects were more pronounced in male wings (15 ± 9.2% reduction) (Fig. 1D) where the L4 vein was lost or fused with L5 due to loss of tissue between the two veins (Fig. 1C, D’, F). In most wings, the L5 vein was considerably thickened in the distal region (Fig. 1D’). At 29 °C, female wings showed more severe wing size reduction (17.4 ± 5.1%) (Fig. S1B) with loss of L4 and PCV (Fig. S1A, B’, E) which were comparable to the male wing phenotypes at 25 °C (Fig. 1C, D, D’, F). Male wings were reduced to 27.4 ± 5.2% of the wild-type size at 29 °C (Fig. S1C, D, F) with a similar loss of L4 (Fig. S1D’). These results indicate that Top2 is required for normal growth and differentiation of the wing. Next, we examined wing imaginal discs from third instar larvae to detect the developmental effects of *Top2 RNAi*. In control normal wing disc (*en* > *GFP/+*), most nuclei stained with DAPI were evenly distributed in the wing pouch (Fig. 2A’, B’). In contrast, wing discs with *Top2 RNAi* showed basal mislocalization of many cell nuclei in the posterior domain (Fig. 2D’). This suggested that *Top2 RNAi* causes cell death, resulting in the basal accumulation of dying cell nuclei. Immunostaining with anti-cleaved Death Caspase-1 (Dcp-1) showed no obvious Dcp-1 staining in wild-type wing discs (Fig. 2B’’). In contrast, wing discs with *Top2 RNAi* had high levels of Dcp-1 staining in the basal region (Fig. 2D”) where fallen nuclei were accumulated (Fig. 2D’). Hence, Top2 is essential for preventing cell death during wing development.

### Top2 knockdown leads to defective eye and head formation

**Fig. 3:**
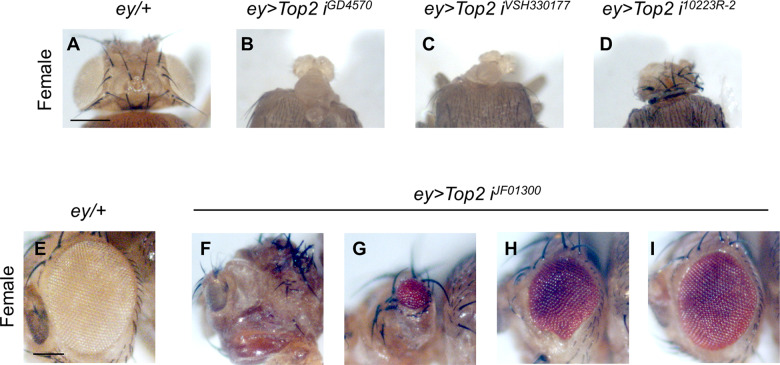
Knockdown of Top2 causes defects in eye and head development. **A**–**D** Knockdown of Top2 in females by three strong RNAi lines using *ey-GAL4* causes pupal lethality at 25 °C. **A** *ey/+* control. **B**–**D** Phenotypes in the head regions from dead pupae. **B** *ey* > *Top2 RNAi*^*GD4570*^. 100% late pupal lethality (*N* = 52). **C** *ey* > *Top2 RNAi*^*VSH330177*^. 100% late pupal lethality (*N* = 46). (**D**) *ey* > *Top2 RNAi*^*10223R-2*^. 94.2% late pupal lethality (*N* = 52) (All three escapers are females). Scale bars in (**A**) and (**E**) are 150 µm. Note that depletion of Top2 in male eye discs by four different RNAi lines (**B**–**D** and *Top2 RNAi*^*JF01300*^) causes pupal lethality with similar defects in the head. **F**–**I** Variable eye phenotypes of *ey* > *Top2 RNAi*^*JF01300*^ adult females. **E** *ey/+* control. **F** no eye (8%, *N* = 50), (**G**) Strong reduction (4%), (**H**) Intermediate reduction (26%), (**I**) Mild reduction (62%). N number of animals.

We examined whether Top2 knockdown causes similar growth defects in the eye. We used *eyeless–GAL4* (*ey-GAL4*) to express *Top2 RNAi* in early eye imaginal disc prior to retinal differentiation. Three *Top2 RNAi* lines (*Top2 i*^*GD4570*^*, Top2 i*^*VSH330177*^*, and Top2 RNAi*^*10223R-2*^) resulted in nearly 100% pupal lethality at 25 °C. Dead pupae from these RNAi lines showed loss of eyes and severe disruption of head structures (Fig. 3B–D).

In contrast, about 32% of *ey* > *Top2 RNAi*^*JF01300*^ flies (*N* = 159) survived to adulthood while the rest died as late pupae. The majority (92%) of these adult flies were females, suggesting that most males could not survive to adulthood. Dead male pupae lost most eye/head structures similar to the phenotype shown in Fig. 3B–D. All surviving female adult flies lost their eyes entirely (Fig. 3F) or showed variable size reduction (Fig. 3G–I). Hence, Top2 is required for the development of the eye and head, and loss of Top2 is more critical for the survival of males than females.

### *Top2* shows synergistic genetic interaction with *Tctp* in organ growth in females

**Fig. 4:**
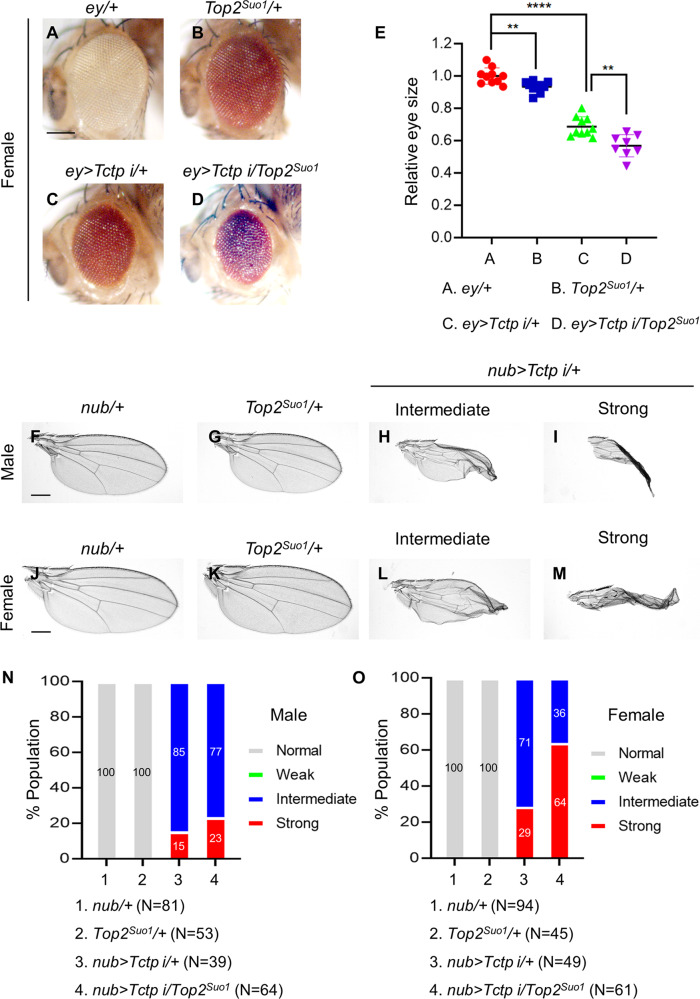
*Top2* shows genetic interaction with *Tctp* in organ growth. **A**–**D** *Top2*^*Suo1*^*/+* enhances *Tctp RNAi* eye phenotype in females. **A** *ey/+* (*n* = 10, x̅ ± s = 1 ± 0.05), (**B**) *Top2*^*Suo1*^*/+* (*n* = 10, x̅ ± s = 0.933 ± 0.034), (**C**) *ey* > *Tctp RNAi/+* (*n* = 10, x̅ ± s = 0.687 ± 0.06), (**D**) *ey* > *Tctp RNAi/Top2*^*Suo1*^ (*n* = 8, x̅ ± s = 0.569 ± 0.069). Scale bar in (**A**) is150 µm. **E** Quantification of relative eye sizes in (**A**–**D**). Statistical analysis in E by unpaired two-tailed student *t*-test, **P < 0.01 and ****P < 0.0001. Error bars in (**E**) are SD. **F**–**M** Wing phenotypes of *Tctp RNAi* and *Top2*^*Suo1*^*/+* in males (**F**–**I**) and females (**J**–**M**). **F**, **J** *nub/+* control, (**G**, **K**) *Top2*^*Suo1*^*/+*, **H**, **L** Intermediate phenotype showing wing size reduction, (**I**, **M**) Strong phenotype showing size reduction with severe folding. Scale bars in (**F**), (**J**) are 300 µm. **N**, **O** Genetic interaction between *Tctp RNAi* and *Top2*^*Suo1*^/+ in males (**N**) and females (**O**). *Top2*^*Suo1*^*/+* weakly enhances the intermediate phenotype to the strong phenotype in males (**N**) while strongly enhancing it in females (**O**). N number of animals.

Since Tctp and Top2 are essential for organ growth, we tested whether Tctp and Top2 are functionally related by using an *ey* > *Tctp RNAi* condition that causes a mild reduction of the eye size at 25 °C (Fig. 4C). As shown in Fig. 3F-I, *ey* > *Top2 RNAi*^*JF01300*^ resulted in variable eye reduction including complete loss of the eye in female adult flies. Double knockdown of both Tctp and Top2 led to pupal lethality in females. Since *Tctp RNAi* or *Top2 RNAi*^*JF01300*^ did not cause significant pupal lethality in females, *Tctp and Top2* seem to synergistically interact in the double RNAi condition.

We also tested their genetic interaction in males. Since *ey* > *Top2 RNAi* caused pupal lethality in males, we used *Top2*^*Suo1*^*/+* heterozygous mutant that has slightly smaller eyes (6.2 ± 5.1%) than male control (Fig. S2A, B, E). Although *Top2*^*Suo1*^*/+* did not significantly enhance the male eye phenotype of *Tctp RNAi* (Fig. S2A-E), it enhanced *Tctp RNAi* eye phenotypes in females (Fig. 4A-E). About 31.3 ± 6% eye size reduction by *Tctp RNAi* was further reduced to 43.1 ± 6.9% by *Top2*^*Suo1*^*/+* (Fig. 4A-E). In addition to the enhanced eye size reduction, *Top2*^*Suo1*^*/+* caused more roughness in the eyes of *Tctp RNAi* in females (Fig. 4D).

Next, we tested genetic interaction between *Tctp* and *Top2* in the wing. *Top2*^*Suo1*^*/+* flies showed normal wings (Fig. 4F, G, J, K). Wing defects were divided into weak reduction, intermediate reduction (Fig. 4H, L), and strong phenotype with more reduction and severe folding (Fig. 4I, M). All of *nub* > *Tctp RNAi* wings were significantly reduced, showing the intermediate phenotype or the strong phenotype (Fig. 4N, O). Double knockdown of Top2 and Tctp showed strongly enhanced wing phenotypes in both sexes (Fig. S2F-M). Because both *Tctp RNAi* and *Top2 RNAi* cause wing defects, we used *Top2*^*Suo1*^*/+*, which does not affect the wing (Fig. 4G, K), to test whether the genetic interaction between *Tctp* and *Top2* is synergistic. *Top2*^*Suo1*^*/+* showed a weak enhancement of the *Tctp RNAi* wing phenotype in males but considerably increased the frequency of strong wing phenotypes in females (Fig. 4F-O). These results support a synergistic genetic interaction of *Top2* with *Tctp* in the females.

### Tctp knockdown reduces Top2 protein levels

Based on the enhancement of *Top2 RNAi* phenotypes by *Tctp RNAi*, Tctp may be involved in the regulation of Top2 function. Hence, we examined whether Top2 levels are affected by Tctp in the wing disc. Immunostaining with anti-Top2 antibody showed nuclear localization of Top2 in all wing disc cells (Fig. S3A’). *Top2 RNAi* driven by *en-GAL4* strongly reduced Top2 staining in the posterior domain of wing disc, confirming the specificity of the anti-Top2 antibody (Fig. S3B’). However, the region near the anterior-posterior (A/P) boundary was not effectively knocked down by *Top2 RNAi*.

**Fig. 5:**
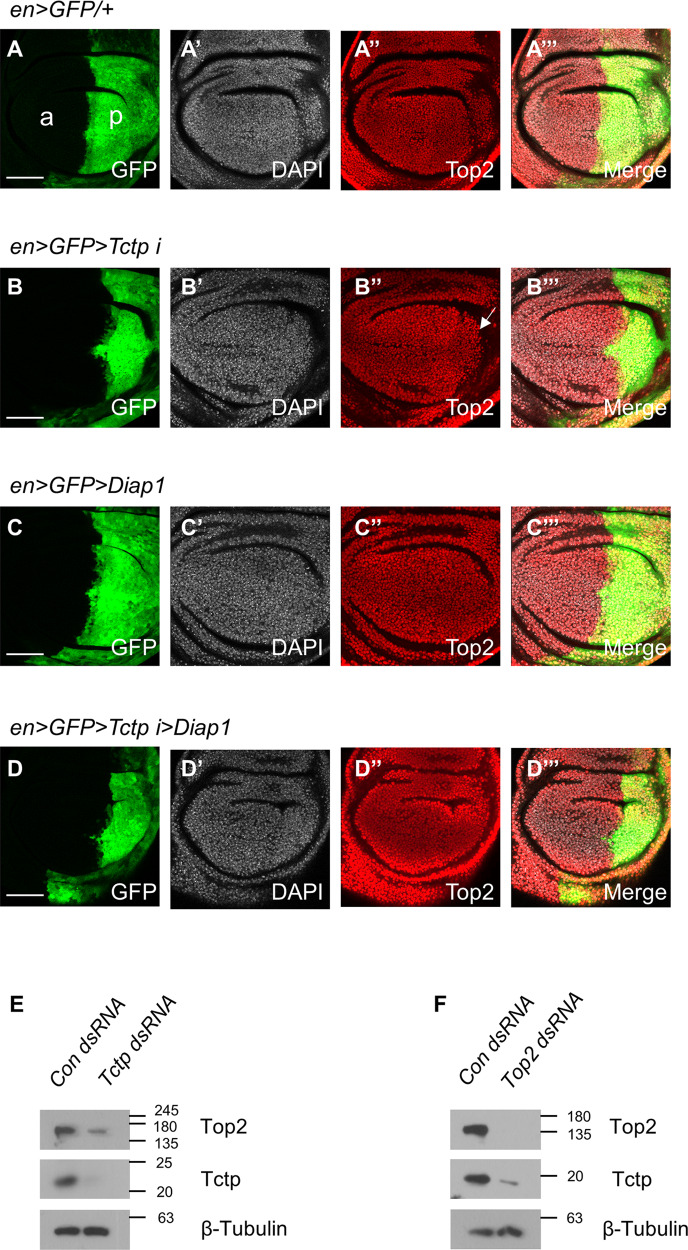
*Tctp RNAi* reduces Top2 protein levels. **A**–**A**’’’ *en* > *GFP/+* shows no significant change in the Top2 level in the posterior domain at 25 °C. **A** GFP, (**A**’) DAPI, (**A**’’) Top2, (**A**’’’) Merge. Scale bars in (**A**–**D**) are 50 µm. **B-B**’’’ *en* > *GFP* > *Tctp i* shows a decrease in Top2 level at 25 °C. (11/14 discs; 78.6%) (**B**) GFP, (**B’**) DAPI, (**B**’’) Top2, (**B**’’’) Merge. The white arrow shows an area of decreased Top2 in the posterior region. **C**–**C**’’’ *en* > *GFP* > *Diap1* shows little change in the Top2 level at 25 °C. (**C**) GFP, (**C**’) DAPI, (**C**’’) Top2, (**C**’’’) Merge. **D**–**D**’’’ *en* > *GFP* > *Tctp i* > *Diap1* shows rescue of Top2 level at 25 °C (8/12 discs; 75%). **D** GFP, (**D**’) DAPI, (**D**’’) Top2, (**D**’’’) Merge. **E**, **F** Western blots stained for Top2, Tctp, and β-Tubulin. (**E**) Top2 protein level is decreased in Tctp-depleted S2 cells (representative of three independent experiments). **F** Tctp protein level is decreased in Top2-depleted S2 cells (representative of three independent experiments).

In wing discs with Tctp knockdown by *en-GAL4*, the posterior region was reduced compared to the anterior region (Fig. 5B–B’’’). Top2 levels were weakly reduced in the posterior region in about 79% of the sample (11/14 discs) (Fig. 5B’’). The reduced Top2 levels in the nuclei of the posterior domain may be related to cell death. Hence, we tested whether inhibition of cell death by Death-associated inhibitor of apoptosis 1 (Diap1) might affect Top2 levels. Diap1 overexpression by *en-GAL4* in the wild-type background did not significantly alter the level of Top2 in the posterior compartment (Fig. 5C–C’’’). However, Top2 reduction was seen in only 25% of the samples (3/12 discs) when Diap1 was overexpressed in the Tctp-depleted posterior region, indicating a partial recovery of the Top2 level (Fig. 5D–D’’’). These results suggest that Top2 reduction by *Tctp RNAi* is at least in part due to cell death.

In a converse experiment, Top2 knockdown by *en-GAL4* caused a local reduction of Tctp levels in the posterior compartment in 100% of discs examined (8/8 discs) (Fig. S4B–B’’’). With Diap1 overexpression, however, only 17% (1/6 discs) of the Top2-depleted wing discs showed Tctp reduction (Fig. S4D–D’’’). Hence, Tctp reduction by *Top2 RNAi* is also related to the caspase activation.

We also tested whether *Tctp RNAi* affects Top2 levels and vice versa in S2 cells. Cells treated with *Tctp RNAi* showed a decrease in Top2 protein level (Fig. 5E). These data suggest that Tctp is required to maintain normal Top2 levels. In addition, *Top2 RNAi* resulted in reduced Tctp levels (Fig. 5F). Taken together, Tctp and Top2 levels are dependent on each other.

Next, we checked whether *Tctp RNAi* affects the level of Top2 at the transcriptional level by Real-time PCR in S2 cells. Our data indicate that about 87% knockdown of Tctp has no significant effect on the Top2 mRNA level. Likewise, a similar knockdown of Top2 did not obviously reduce the Tctp mRNA level (Fig. S5). Hence, the mutual regulation between Tctp and Top2 is likely to occur at the post-transcriptional level.

### Top2 overexpression impairs organ growth

Our data thus far indicate that Top2 is required for the proper growth of organs. To test whether Top2 overexpression can promote organ growth, we used a transgenic line overexpressing Top2. Top2 overexpression by *en-GAL4* was confirmed by a strong enhancement of Top2 immunostaining in the posterior wing disc (Fig. S6A’, B’). Interestingly, Top2 overexpression at 25 °C resulted in a reduction of wing size in males (21.3 ± 7.4%) (Fig. S6C–E) and females (11.9 ± 6.0%) (Fig. S6F–H). We also checked whether Top2 overexpression can impair eye development. Top2 overexpression by *ey-GAL4* resulted in weakly reduced eyes (Fig. S6I–N).

In contrast to relatively weak phenotypes of Top2 overexpression driven by *en-GAL4* or *ey-GAL4*, we found more severe wing growth defects by Top2 overexpression in the entire wing region by *nub-GAL4* (Fig. S7A–H). Both females and males of *nub* > *Top2-1* died at 29 °C. To further support the effects of Top2 overexpression, we tested four additional independent *UAS-Top2* transgenic lines. All *UAS-Top2* lines were generated using PhiC31 transgenesis for targeted insertion of *UAS-Top2* at the same genomic site (28E7 on the second chromosome). 47–75% of female flies overexpressing Top2 showed strong defects with size reduction and wrinkling (Fig. S7G). The wings of the remaining flies were nearly normal. In males, 78–92% of flies showed similar reduction and wrinkling in the wing (Fig. S7H). The reduction of organ size by either knockdown or overexpression of *Top2* suggests that proper regulation of its level is required for the normal growth of organs.

### Top2 overexpression can partially rescue *Tctp RNAi* phenotype

**Fig. 6:**
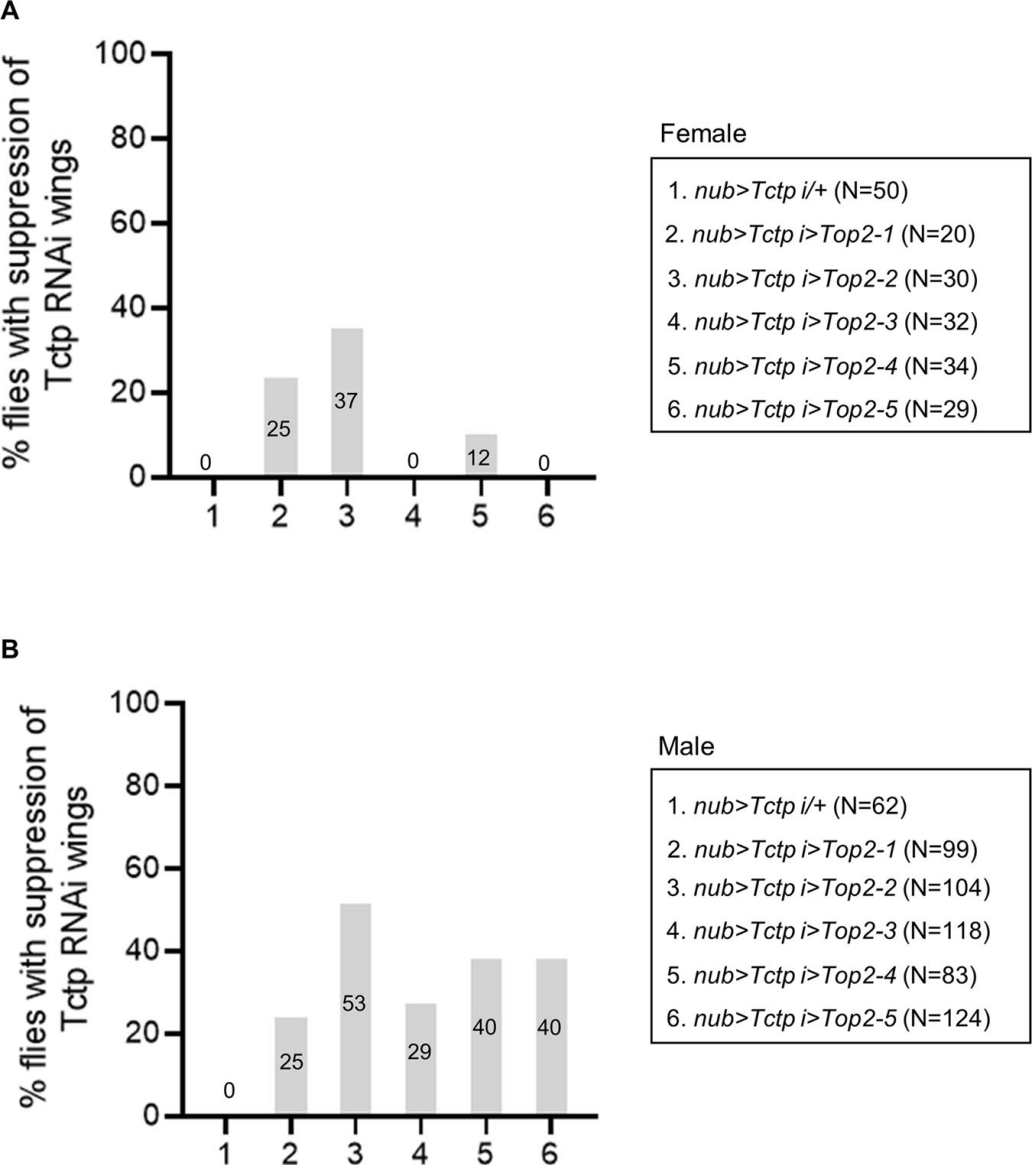
Overexpression of Top2 can partially suppress the *Tctp RNAi* phenotype. **A**, **B** Genetic interaction between *Tctp RNAi* and Top2 overexpression by *UAS-Top2* lines (*UAS-Top2-1* to *UAS-Top2-5*). **A** Females. Control progeny (*nub* > *Tctp i/+*) from a cross between *nub* > *Tctp RNAi* and *w*^*1118*^ flies shows 100% wing defects with no suppression. *Tctp RNAi* phenotypes are suppressed by Top2 overexpression from three out of five *UAS-Top2* lines (lines 2, 3, and 5) (**B**) Males. Control progeny (*nub* > *Tctp i/+*) shows 100% wing defects with no suppression. *Tctp RNAi* phenotypes are suppressed by Top2 overexpression from all five *UAS-Top2* lines. N number of animals. Numbers in the bars indicate % adults showing the suppression.

We have shown synergistic enhancement of wing phenotypes when loss-of-function conditions for Tctp and Top2 are combined (Fig. 4O). For further characterization of the relationship between these two genes, we tested genetic interaction between *Tctp RNAi* and Top2 gain-of-function. Because Top2 overexpression itself impairs wing development (Fig. S7A–H), it may additively enhance the *Tctp RNAi* phenotype. However, since *Tctp RNAi* causes a reduction in the Top2 level (Fig. 5B-B’’’, E), a proper level of Top2 overexpression may also lead to rescue of *Tctp RNAi* phenotype. To test this possibility, we examined whether the wing phenotypes of *Tctp RNAi* shown in Fig. 4H, I, L, and M can be suppressed to near normal wings by Top2 overexpression. In a control cross, *nub* > *Tctp RNAi/+* progeny showed 100% penetration of the wing defects in both sexes. Top2 overexpression from three of five *UAS-Top2 lines* (1, 2, and 4) led to suppression of the *Tctp RNAi* phenotype in approximately 12–37% female wings examined (Fig. 6A). In male wings, the suppression was found in approximately 25–53% of wings from all five *UAS-Top2* lines (Fig. 6B). Because the rate of suppression was higher in males, we tested whether Top2 overexpression might differentially affect the level of Tctp in a sex-dependent manner. However, Tctp levels were not noticeably altered in male or female wing discs by Top2 overexpression (Fig. S8A–D’’’) suggesting that the difference is probably not due to sex-specific regulation of Tctp levels by Top2.

## Discussion

In this study, we provided evidence for a positive functional relationship between *Tctp* and *Top2* in *Drosophila* organ development. Our data show that the targeted knockdown of Top2 in imaginal discs causes apoptosis and disrupts organ development. Interestingly, *Top2 RNAi* organ phenotypes and lethality are influenced by sex. In *Drosophila*, dosage compensation of the male X chromosome is mediated by the Male-specific lethal (MSL) complex [52–55]. It has been shown that Top2 is recruited to the MSL complex to enhance the transcription level of X-linked genes [44]. Hence, the stronger *Top2 RNAi* phenotypes in males may be related to defective dosage compensation in males.

Our data show that *Tctp RNAi* phenotypes are strongly enhanced in female *Top2*^*Suo1*^*/+* heterozygotes, implying a synergistic genetic interaction between the two genes. In addition, *Tctp RNAi* reduces the level of Top2 in developing wing discs. Similarly, *Top2 RNAi* leads to a reduction of Tctp levels in wing discs. Significant numbers of Tctp- or Top2-depleted cells are probably lost during wing development based on the reduced size of adult wings. Our data show that overexpression of Diap1 partially suppresses the reduction of Tctp/Top2 levels in wing discs. Hence, the decreased protein levels in the targeted region of the wing disc might be in part due to cell death signaling, albeit not necessarily due to an absence of cells at the 3^rd^ instar larval stage. In S2 cells, *Tctp RNAi* also reduces Top2 levels and vice versa, despite the presence of normal levels of β-Tubulin. Hence, the altered levels of Tctp and Top2 may not be entirely due to cell death. It is currently unknown how Tctp and Top2 affect their levels. One possibility is that Tctp may be involved in transcriptional activation of the *Top2* gene because Tctp plays a role in gene regulation by directly interacting with Brahma (Brm)/Swi/SNF chromatin remodeler [39]. However, our real-time PCR data (Fig. S5) suggest that the mutual regulation between Tctp and Top2 is not at the transcriptional level. Since human TCTP and TOP2 physically interact, Tctp and Top2 may post-transcriptionally regulate their protein stability by forming a complex.

Interestingly, we noticed that the Top2 level was not affected by *Top2 RNAi* (Fig. S3B’) in the region near the anterior-posterior (A/P) boundary. This phenomenon is not due to a defect in the *en-GAL4* driver because the GFP reporter for *en-GAL4* was correctly induced in the entire posterior domain, including the boundary region (Fig. S3B). However, the A/P boundary region may be influenced by a non-autonomous effect from the anterior compartment or by an unknown feedback mechanism to compensate for the loss of Top2.

We have shown that either reduction or overexpression of Top2 causes severe growth defects in the wing. This suggests that Top2 protein must be maintained at an optimal level for proper control of organ growth. Mammalian TOP2 is known to be required for the survival of proliferating cells by generating transient DNA strand breaks in various DNA processes, but it is also potentially cytotoxic because the accumulation of breaks can cause DNA aberrations such as translocation, leading to apoptosis [56]. Hence, the observed defects in organ growth by Top2 overexpression may be due to excessive DNA damages.

An important question is whether the regulation of the Top2 level by Tctp is functionally significant. To address this question, we checked whether Top2 overexpression can suppress the *Tctp RNAi* phenotype by overriding the loss of Top2. Indeed, a significant fraction of the flies tested shows suppression of the *Tctp RNAi* wing phenotype under the Top2 overexpression condition (Fig. 6A, B), implying a physiological role of the Tctp-dependent regulation of Top2 levels. Although the suppression of the *Tctp RNAi* phenotype by Top2 overexpression is seen in both sexes, male flies show higher rates of suppression than females. Top2 overexpression has no significant effects on Tctp levels in wing discs of either males or females (Fig. S8A-D’’’). Hence, sex-dependent effects of Top2 overexpression are probably not due to differential regulation of Tctp levels in males and females. Further studies are necessary to understand the basis for sex-dependent effects of Top2. Since Tctp and Top2 are evolutionarily conserved proteins, it would also be interesting to see whether mammalian TCTP and TOP2 are mutually regulated to maintain their levels in developing tissues and organs as seen in *Drosophila*.

## Supplementary information

Supplementary legend

Figure S1

Figure S2

Figure S3

Figure S4

Figure S5

Figure S6

Figure S7

Figure S8

## Data Availability

All data generated or analyzed during this study are included in this published article and its supplementary information files.
