# Supplementary material for: Topoisomerase II is regulated by translationally controlled tumor protein for cell survival during organ growth in Drosophila
Source: Cell Death Dis. 2021 Aug 27;12(9):811. doi: 10.1038/s41419-021-04091-y (PMC8397738; doi:10.1038/s41419-021-04091-y)
Supplement: Supplementary file 1 — Supplementary legend [file 41419_2021_4091_MOESM1_ESM.docx]

**Supplementary Figure legends**

**Fig. S1. Knockdown of Top2 causes severe wing reduction at 29^°^C.**

(A, B) Effects of *Top2 RNAi* by *en-GAL4* in females at 29^°^C. The A/P boundary is indicated by a dotted line. (A) *en>GFP/+* (n=11, x̅±s=1±0.028), (B) *en>GFP>Top2 RNAi^JF01300^* (n=15, x̅±s=0.826±0.051). Scale bars are 300 µm. (A’, B’) A magnification of (A, B). Veins 4 (L4) and 5 (L5) appear to be fused. Wing cells near the vein are irregular and abnormal.

(C, D) Effects of *Top2 RNAi* by *en-GAL4* in males at 29^°^C. (C) *en>GFP/+* (n=13, x̅±s=1±0.057). (D) *en>GFP>Top2 RNAi^JF01300^* (n=7, x̅±s=0.726±0.052). (C’, D’) A magnification of (C, D).

(E) Quantification of relative wing sizes in A, B. Statistical analysis in E, F by unpaired two-tailed student t-test, *****P*<0.0001. Error bars in E, F are SD.

(F) Quantification of relative wing sizes in C, D.

**Fig. S2. Genetic interaction between *Tctp RNAi* and *Top2^Suo1^/+* or *Top2 RNAi*.**

(A-D) No significant genetic interaction between *Tctp RNAi* and *Top2^Suo1^/+* in male. (A) *ey/+* (n=10, x̅±s=1±0.051), (B) *Top2^Suo1^/+* (n=10, x̅±s=0.938±0.051)*,* (C) *ey>Tctp RNAi/+* (n=10, x̅±s=0.719±0.097), (D) *ey>Tctp RNAi/Top2^Suo1^* (n=12, x̅±s=0.725±0.102). Scale bar in A is 150 µm.

(E) Quantification of relative eye sizes in A-D. Statistical analysis in E by unpaired two-tailed student t-test, n.s. (*P*>0.05), **P*<0.05, and *****P*<0.0001. Error bars in E are SD.

(F-I) Genetic interaction between *Top2 RNAi* and *Tctp RNAi* in females at 25^°^C. (F) *nub/+*, (G) *nub>Top2 i^JF01300^* (N=32; 43.8% penetrance), (H) *nub>Tctp i/+* (N=33; 100%), (I) *nub>Tctp i>Top2 i^JF01300^* (N=29; 100%). Scale bars in F, J are 300 µm.

(J-M) Genetic interaction between *Top2 RNAi* and *Tctp RNAi* in males at 25^°^C. (J) *nub/+*, (K) *nub>Top2 i^JF01300^* (N=27; 100%), (L) *nub>Tctp i/+* (N=29; 100%), (M) *nub>Tctp i>Top2 i^JF01300^* (N=21; 100%). N: number of animals

**Fig. S3. Validation of Top2 antibody using *Top2 RNAi* wing discs.**

Wing discs were stained for GFP (A and B), Top2 (A’ and B’), and DAPI (A’’ and B’’) as indicated in each panel. A’’’ and B’’’ are merged images.

(A-A’’’) *en>GFP/+* shows little change in Top2 staining in the posterior region at 25°C. Yellow dotted lines in A’-B’’ indicate the A/P boundary. Scale bars in A, B are 50 µm.

(B-B’’’) *en>GFP>Top2 RNAi^JF01300^* shows decreased Top2 staining in the posterior region except for the A/P boundary region at 25°C (3/3 discs; 100%).

**Fig. S4. Tctp reduction by *Top2 RNAi* is partially restored by Diap1 overexpression.**

(A-A’’’) *en>GFP/+* shows no change of Tctp2 level at 29°C. (A) GFP, (A’) DAPI, (A’’) Tctp, (A’’’) Merge. Yellow dotted lines in A’-D’’ indicate the A/P boundary. Scale bars in A-D are 50 µm.

(B-B’’’) *en>GFP>Top2 i* shows decreased Tctp levels at 29°C. (8/8 discs; 100%) (B) GFP, (B’) DAPI, (B’’) Tctp, (B’’’) Merge. White arrows show decreased Tctp (B’’) and condensed GFP staining (B) in the posterior region.

(C-C’’’) *en>GFP>Diap1* shows no change of Tctp level at 29°C. (C) GFP, (C’) DAPI, (C’’) Tctp, (C’’’) Merge.

(D-D’’’) *en>GFP>Top2 i>Diap1* shows a partial rescue of Tctp level at 29°C. (5/6 discs; 83.3%) (D) GFP, (D’) DAPI, (D’’) Tctp, (D’’’) Merge.

**Fig. S5. Tctp and Top2 do not regulate each other at the transcription level.**

Quantification of transcription from Tctp/Top2 dsRNA-treated S2 cells by real-time PCR. Tctp dsRNA treatment efficiently downregulated Tctp expression (Tctp#1: N=3; m±sem=0.126±0.051, Tctp#2: N=3; m±sem=0.133±0.051) but not Top2 expression (Top2#1: N=3; m±sem=0.868±0.085, Top2#2: N=3; m±sem=0.922±0.096). Top2 dsRNA treatment decreased Top2 expression (Top2#1: N=3; m±sem=0.128±0.012, Top2#2: N=3; m±sem=0.132±0.015) but did not significantly affect Tctp expression (Tctp#1: N=3; m±sem=0.930±0.024, Tctp#2: N=3; m±sem=0.980±0.0479). Multiple T-test was used for statistical analysis and statistical significance was determined using the Holm-Sidak method, with alpha = 0.05. n.s (*P*>0.05), ****P*<0.001, and *****P*<0.0001. N: the number of independent experiments. m: average of relative expression levels.

**Fig. S6. Validation and characterization of Top2 overexpression.**

(A-A’’’) The apical section of *en>GFP>Top2-1* wing disc shows increased Top2 in the posterior region of the wing disc at 25°C (4/4 discs; 100%). (A) GFP, (A’) Top2, (A’’) DAPI, (A’’’) Merge. Yellow dotted lines in A’-B’’ indicate the A/P boundary. Scale bars in A, B are 50 µm.

(B-B’’’) Basal section of the same disc shown in (A-A’’’). Ectopic Top2 staining is seen in the posterior basal region of the wing pouch.

(C, D) Overexpression of Top2 shows smaller wing in male, 25°C. (C) *en>GFP/+* (n=11, x̅±s=1±0.040), (D) *en>GFP>Top2-1* (N=31; 100%, n=14, x̅±s=0.787±0.074). Scale bars in C, F are 300 µm.

(E) Quantification of relative wing sizes in C, D. Statistical analysis in E, H, K, N by unpaired two-tailed student t-test, ****P*<0.001, and *****P*<0.0001. Error bars in E, H, K, N are SD.

(F, G) Overexpression of Top2 shows smaller wing in female, 25°C. (F) *en>GFP/+* (n=10, x̅±s=1±0.024), (G) *en>GFP>Top2-1* (N=31; 100%, n=10, x̅±s=0.881±0.060),

(H) Quantification of relative wing sizes in F, G.

(I, J) Overexpression of Top2 shows slightly smaller eye in female, 25°C. Scale bars in I, L are 150 µm. (I) *ey/+* (n=10, x̅±s=1±0.031), (J) *ey>Top2-1* (n=10, x̅±s=0.894±0.034).

(K) Quantification of relative eye sizes in I, J.

(L, M) Overexpression of Top2 shows slightly smaller eye in male, 25°C. (L) *ey/+* (n=10, x̅±s=1±0.034), (M) *ey>Top2-1* (n=9, x̅±s=0.938±0.027).

(N) Quantification of relative eye sizes in L, M. N: number of animals

**Fig. S7. Top2 overexpression impairs wing development.**

(A-F) Wing phenotypes of Top2 overexpression in females (B, C) and males (E, F). (A, D) *nub/+* control, (B, E) Nearly normal with slight size reduction, (C, F) Strong phenotypes showing severe reduction and wrinkling. Scale bars are 300 µm.

(G, H) Quantification of wing phenotypes by Top2 overexpression in females (G) and males (H). N: number of animals

**Fig. S8. Effects of Top2 overexpression on Tctp levels in the wing disc.**

(A-A’’’) A wing disc of *en>GFP/+* at 29°C in males. (A) GFP, (A’) DAPI, (A’’) Tctp, (A’’’) Merge. Yellow dotted lines in A’-D’’ indicate the A/P boundary. Scale bars in A-D are 50 µm.

(B-B’’’) *en>GFP>Top2-1* shows little change of Tctp level in the posterior region at 29°C in males. (8/8 discs; 100%) (B) GFP, (B’) DAPI, (B’’) Tctp, (B’’’) Merge.

(C-C’’’) A wing disc of *en>GFP/+* at 29°C in females. (C) GFP, (C’) DAPI, (C’’) Tctp, (C’’’) Merge.

(D-D’’’) *en>GFP>Top2-1* shows little change of Tctp level in the posterior region at 29°C in females. (6/8 discs; 75%) (D) GFP, (D’) DAPI, (D’’) Tctp, (D’’’) Merge.
