# Supplementary figures and images for: Topoisomerase II is regulated by translationally controlled tumor protein for cell survival during organ growth in Drosophila
Source: Cell Death Dis. 2021 Aug 27;12(9):811. doi: 10.1038/s41419-021-04091-y (PMC8397738; doi:10.1038/s41419-021-04091-y)

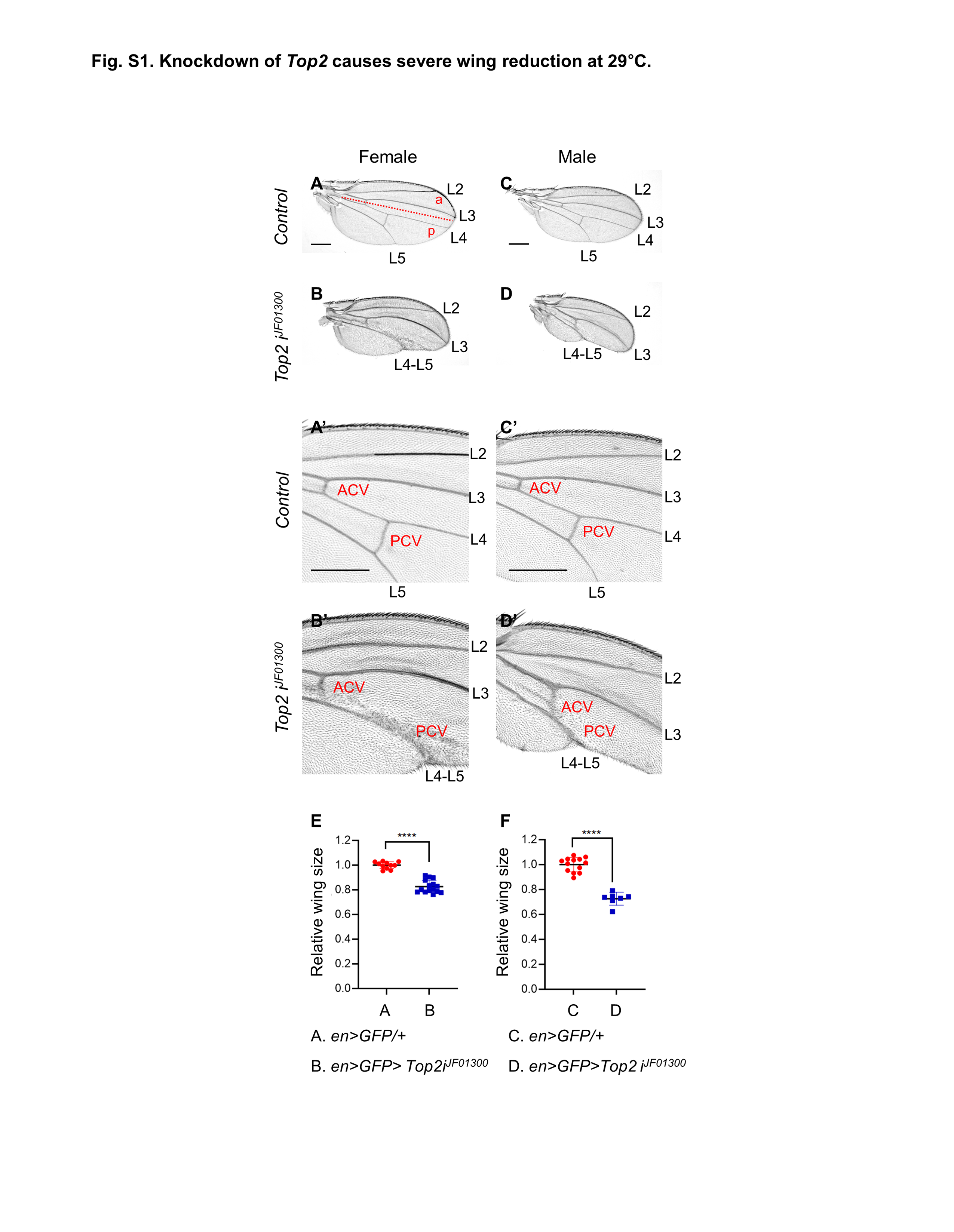

Supplement: Supplementary file 2 — Figure S1 [file 41419_2021_4091_MOESM2_ESM.tif]

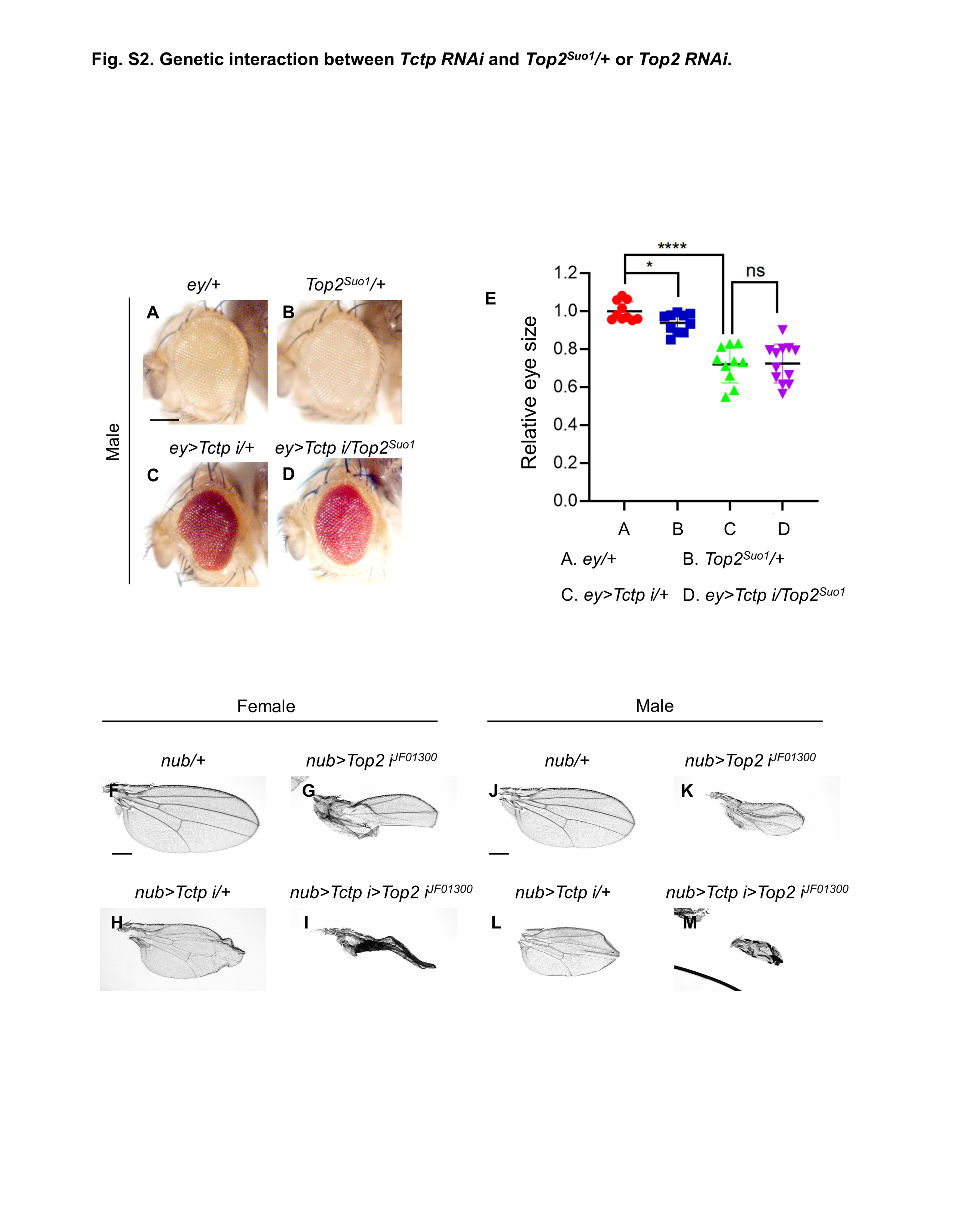

Supplement: Supplementary file 3 — Figure S2 [file 41419_2021_4091_MOESM3_ESM.tif]

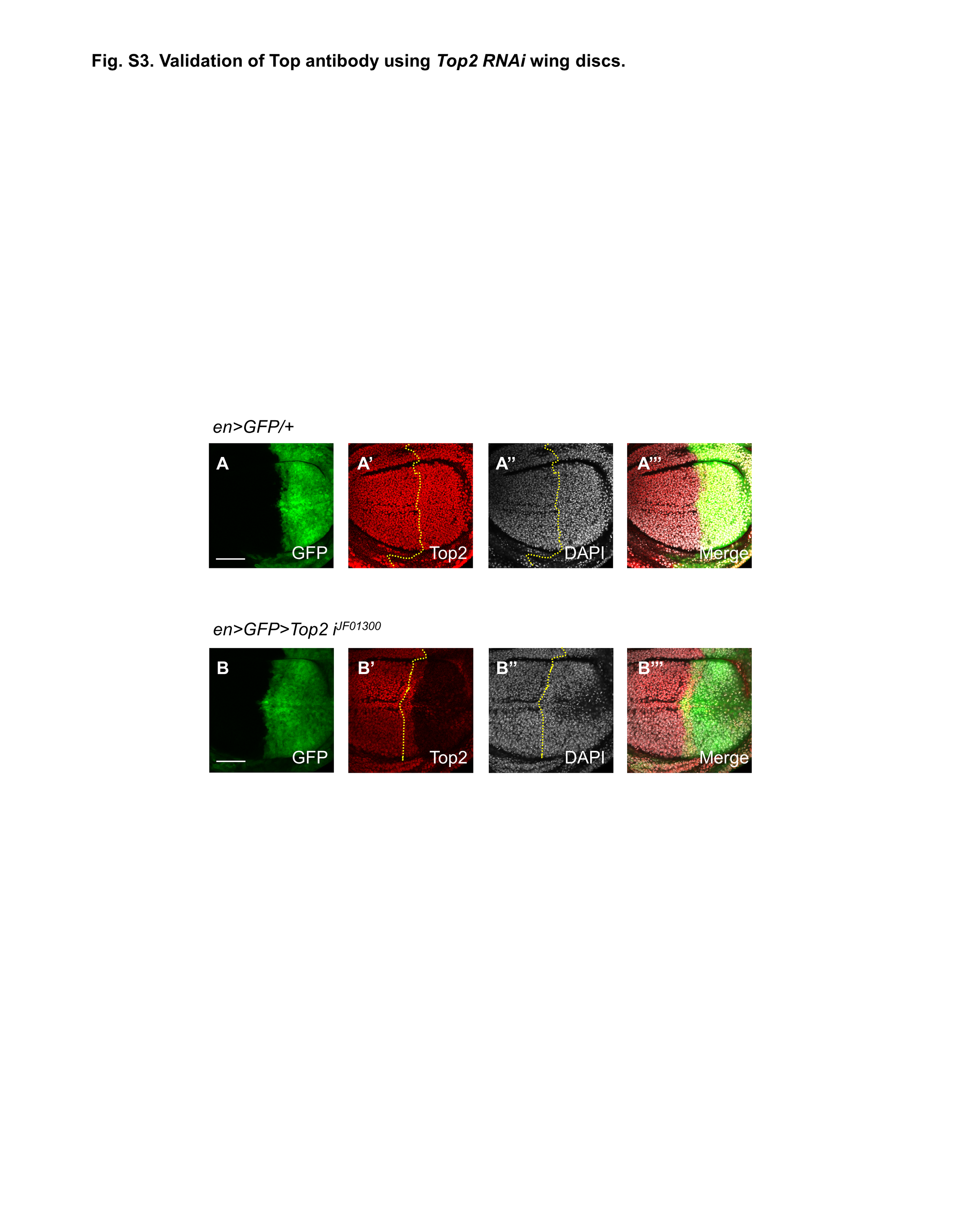

Supplement: Supplementary file 4 — Figure S3 [file 41419_2021_4091_MOESM4_ESM.tif]

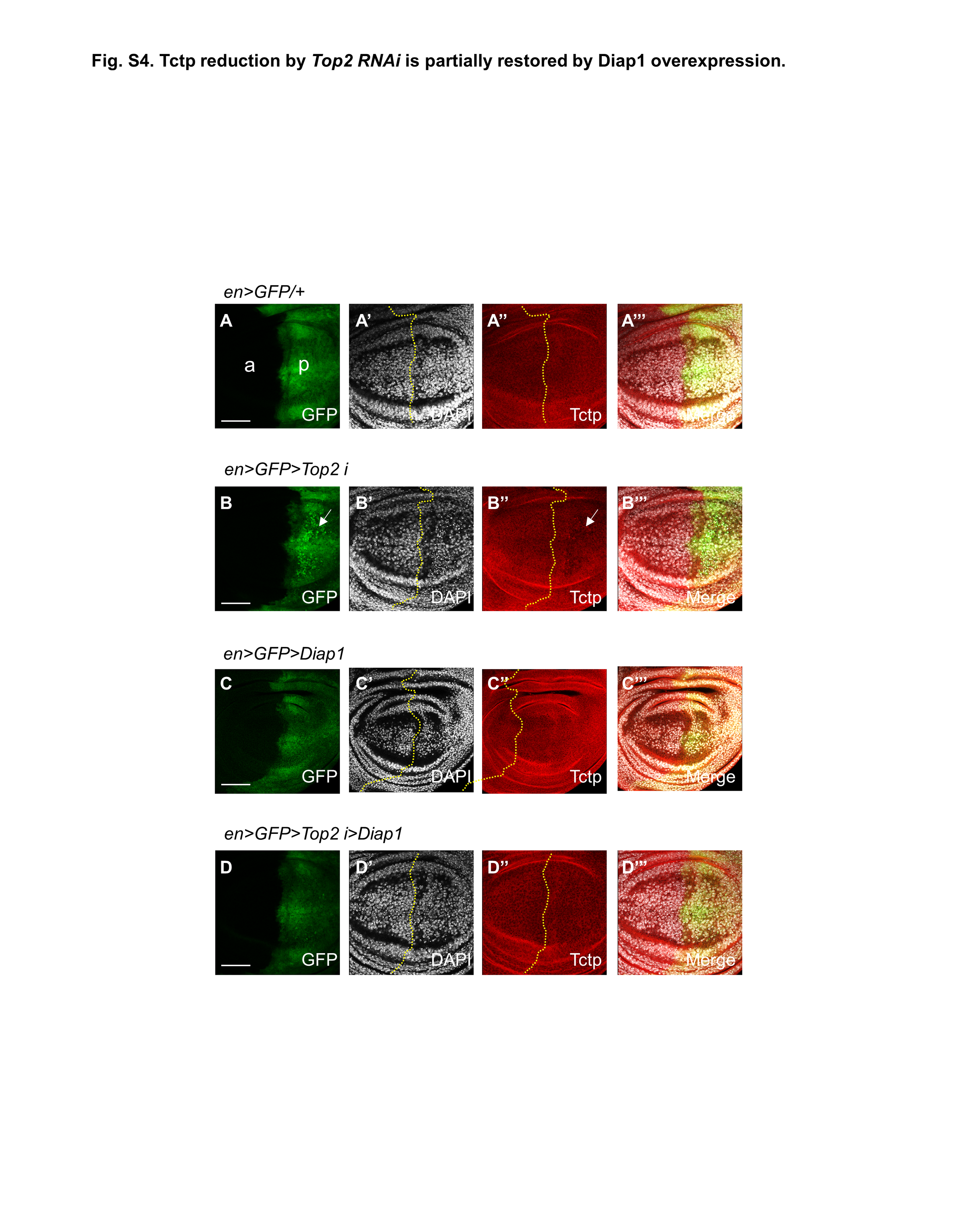

Supplement: Supplementary file 5 — Figure S4 [file 41419_2021_4091_MOESM5_ESM.tif]

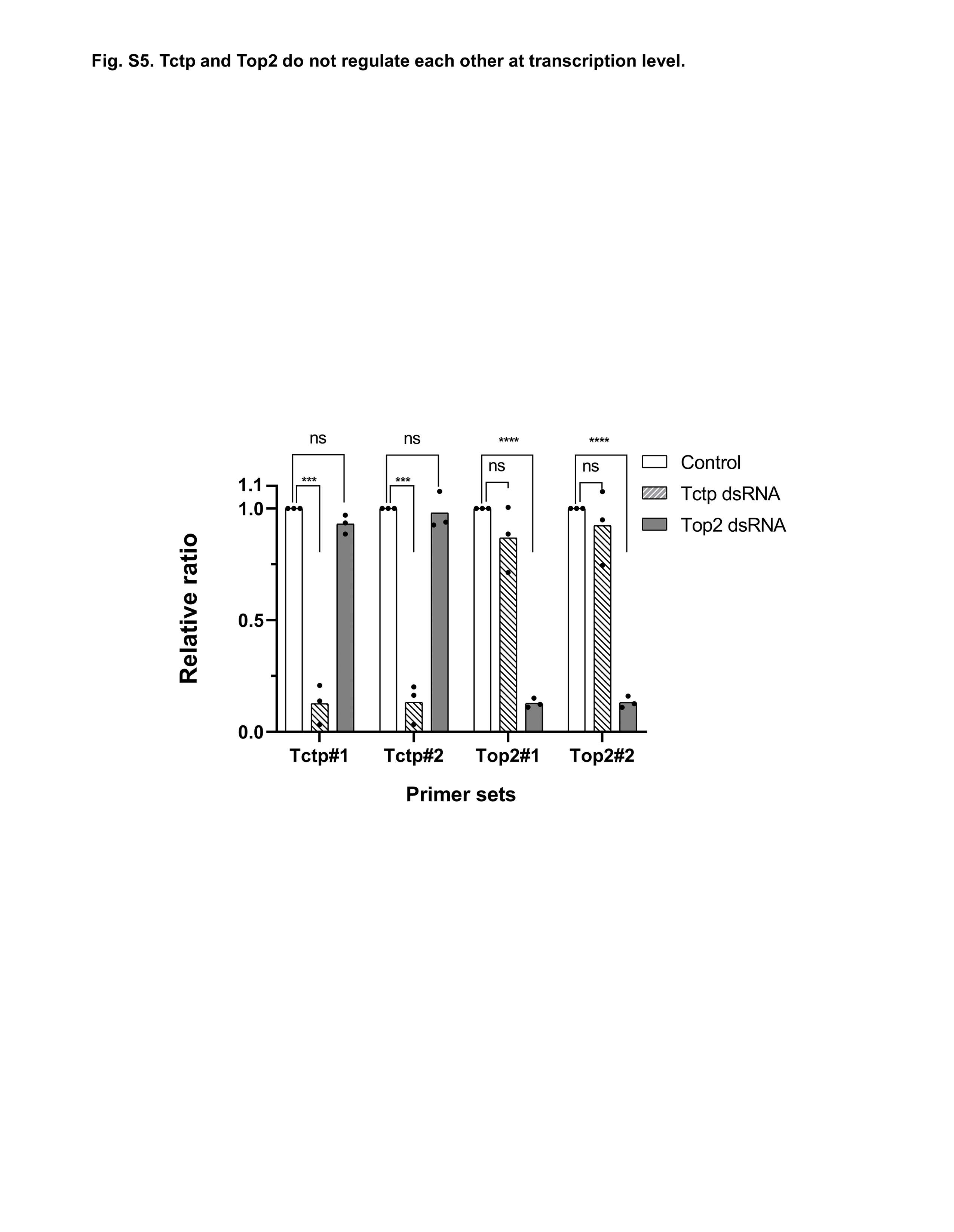

Supplement: Supplementary file 6 — Figure S5 [file 41419_2021_4091_MOESM6_ESM.tif]

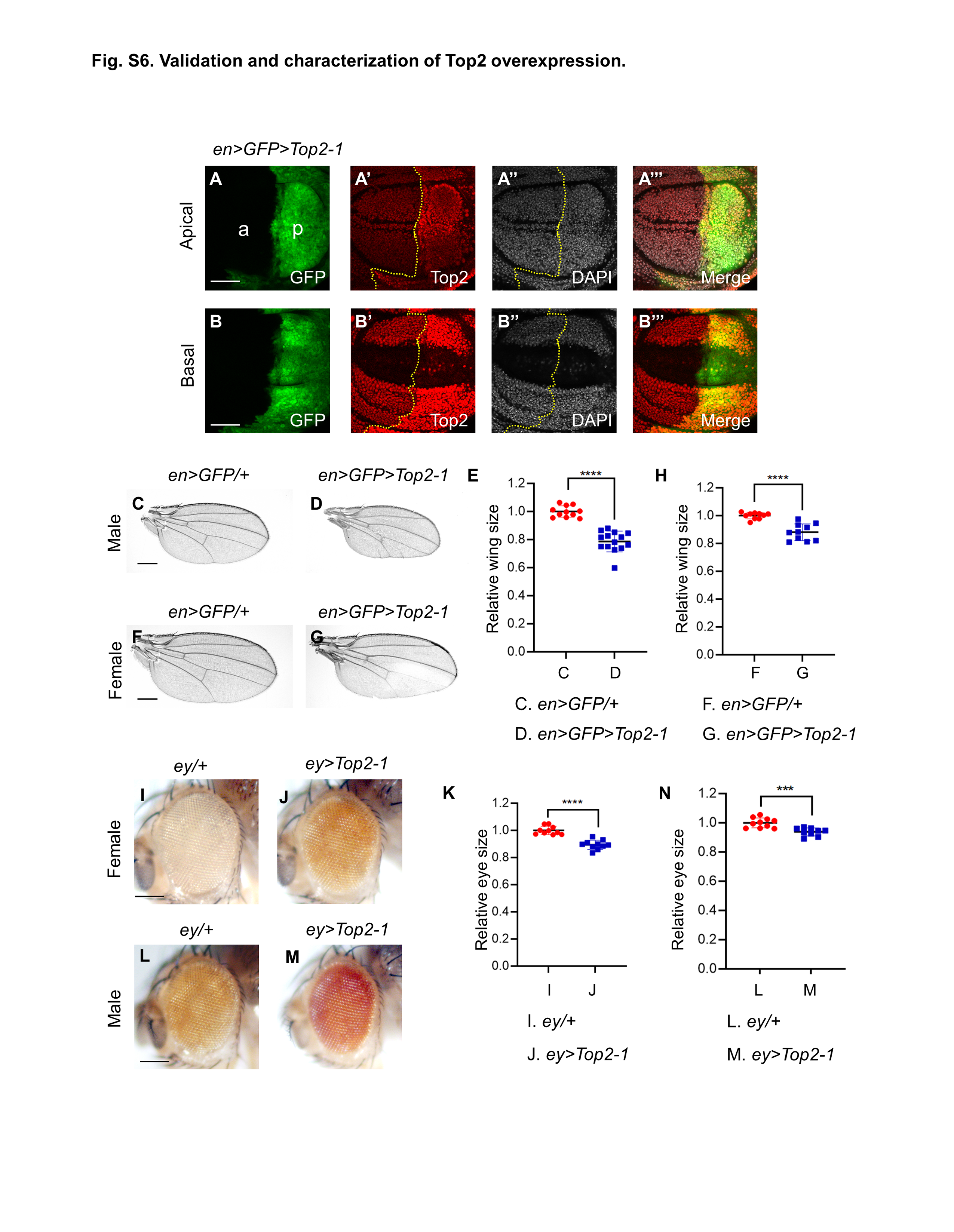

Supplement: Supplementary file 7 — Figure S6 [file 41419_2021_4091_MOESM7_ESM.tif]

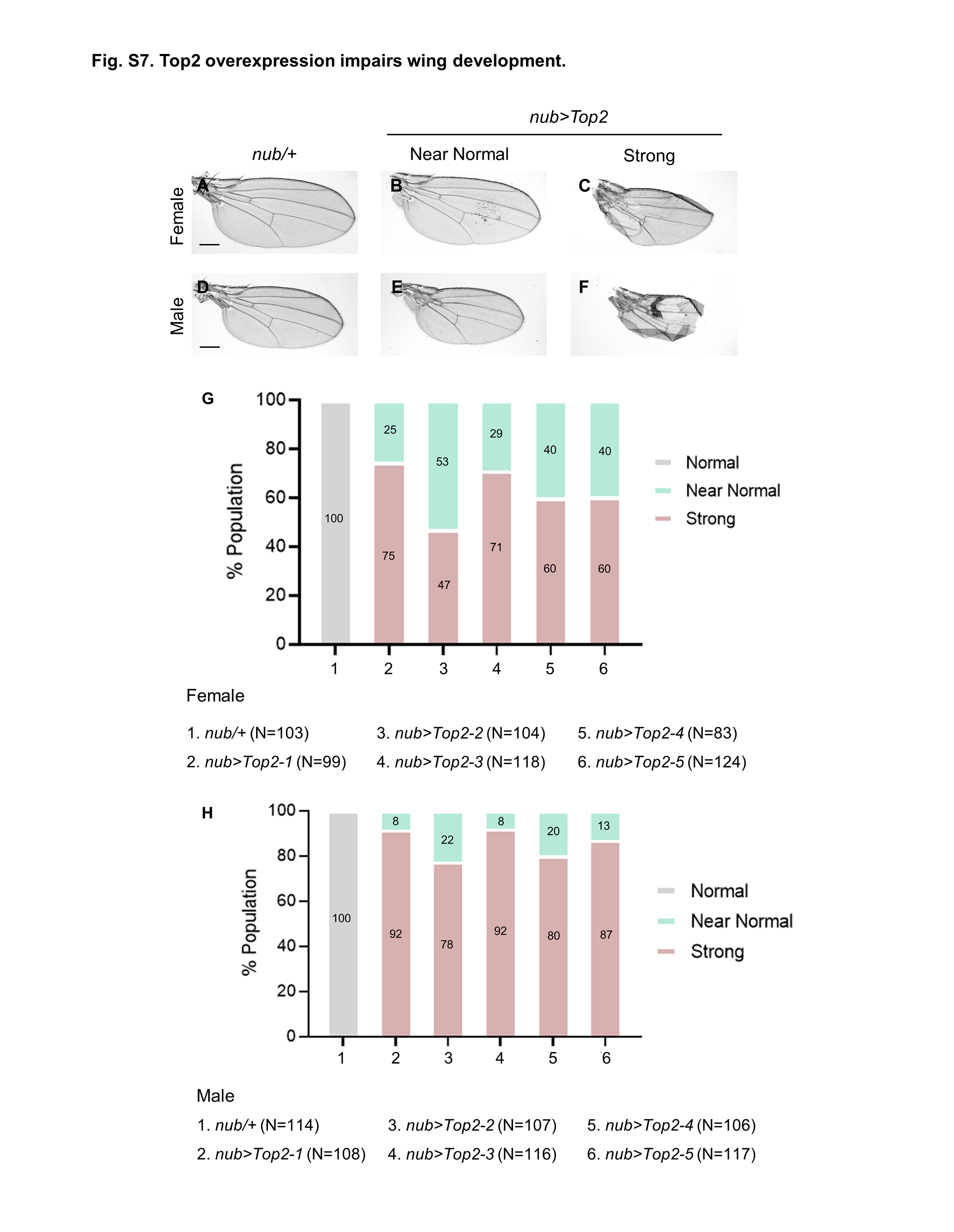

Supplement: Supplementary file 8 — Figure S7 [file 41419_2021_4091_MOESM8_ESM.tif]

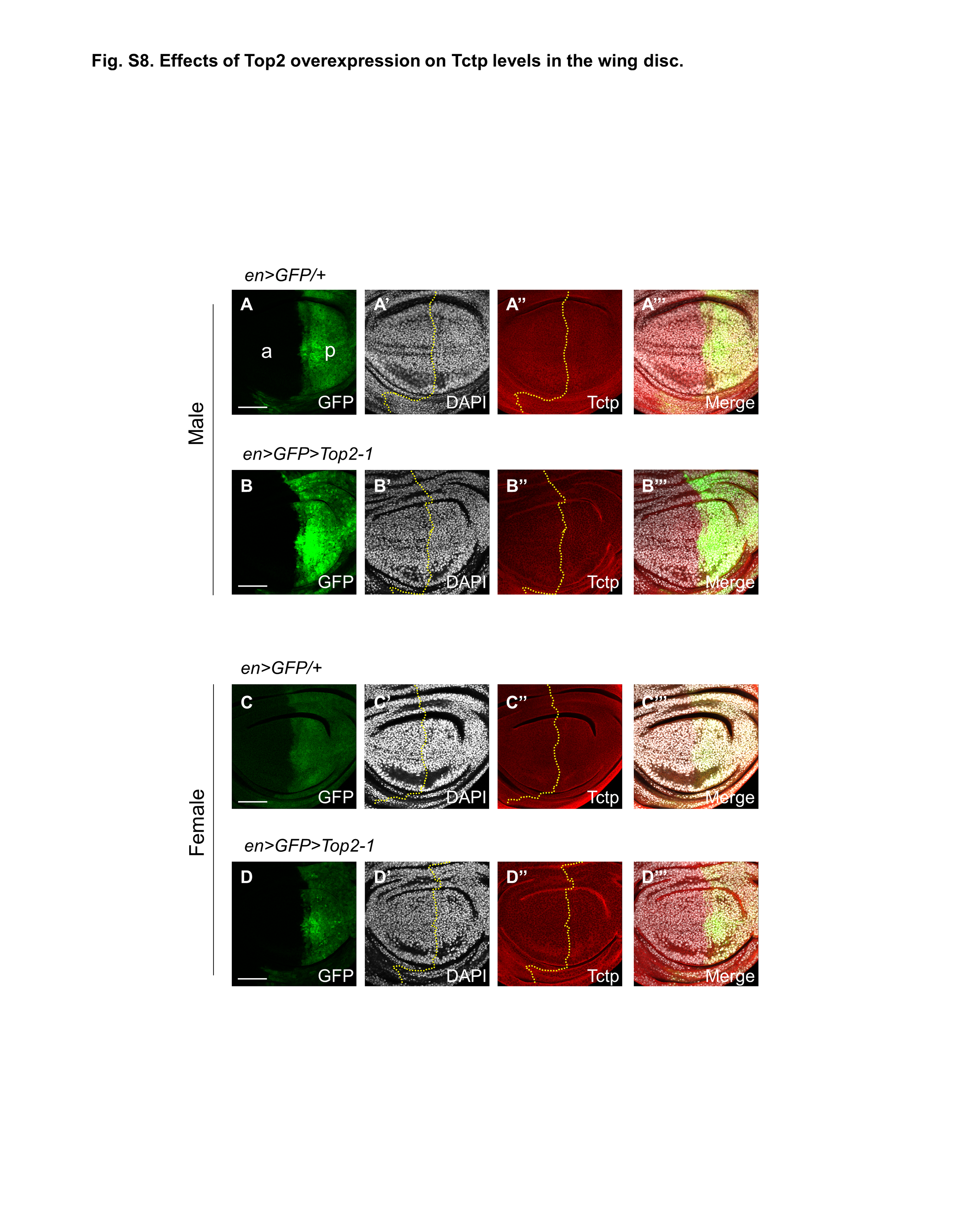

Supplement: Supplementary file 9 — Figure S8 [file 41419_2021_4091_MOESM9_ESM.tif]
